# Combining Dynamic Network Analysis and Cerebral Carryover Effect to Evaluate the Impacts of Reading Social Media Posts and Science Fiction in the Natural State on the Human Brain

**DOI:** 10.3389/fnins.2022.827396

**Published:** 2022-02-21

**Authors:** Bo Hu, Yu-Ling Cui, Ying Yu, Yu-Ting Li, Lin-Feng Yan, Jing-Ting Sun, Qian Sun, Jing Zhang, Wen Wang, Guang-Bin Cui

**Affiliations:** ^1^Department of Radiology, Functional and Molecular Imaging Key Lab of Shaanxi Province, Tangdu Hospital, Fourth Military Medical University Air Forced Medical University, Xi’an, China; ^2^Department of Radiology, The First Affiliated Hospital, Xi’an Jiaotong University, Xi’an, China

**Keywords:** fMRI, dynamic functional network connectivity, carryover effect, social media, science fiction

## Abstract

Social media has been associated with decreased attention, memory, and learning abilities; however, the underlying mechanisms remain unclear. Dynamic function network connectivity (dFNC) analysis is suitable for uncovering dynamical brain activity. Besides, the effects of a cognitive task may persist for a while on the brain, even after the termination of the task, also known as the carryover effect. Consequently, we combined the dFNC analysis and cerebral carryover effects to study the brain dynamics of reading social media posts in the natural state and comparatively investigated the brain dynamics of reading science fiction on the smartphone. We performed functional MRI (fMRI) scans of all subjects at baseline and then assigned them a social media post or science fiction reading task. Immediately after, another fMRI scanning was performed for these subjects. We found that the change between dFNC states, the number of dFNC states, and the total distances increased after reading science fiction. Furthermore, the global, local, and nodal efficiencies of the deep-thinking state tended to increase after reading science fiction. On reading social media posts, the functional connectivity (FC) between the default mode network (DMN) and bilateral frontoparietal network (FPN) decreased, while the FC between DMN and visual network (VN) increased. Given the current evidence, we concluded that reading science fiction could substantially increase brain activity and network efficiency, while social media was related to abnormal FCs between DMN, VN, and FPN.

## Introduction

With the advances in the Internet, social media has become a pervasive component of our daily lives; Twitter and Facebook are the most used social networking sites (Kuss and Griffiths, 2017). For several individuals, social media has become an important leisure activity as it allows individuals to connect online regardless of time and space constraints. However, previous studies show that social media is associated with decreased attention, memory, and learning abilities (Crone and Konijn, 2018; Firth et al., 2019), along with poor performance in text comprehension relative to the print-based medium (Annisette and Lafreniere, 2016; Clinton, 2019). However, there is no conclusive evidence underlying these cognitive deficits due to the usage of social media. Thus, brain activity when reading social media posts should be investigated to elucidate the underlying neural mechanisms causing these deficits (Meshi et al., 2015).

Functional MRI (fMRI) is commonly used to study functional brain activity. In recent years, researchers have shown great interest in dynamic functional network connections (dFNCs) and consequently found that dFNC may bring in more efficient information than the static ones. In addition, considering that the brain is in a dynamic state while reading social media posts, dFNC analysis is more suitable to study the dynamics of brain activity. This method is robust and has been verified in several previous studies, including those involving the migraines (Tu et al., 2019), autism (Li et al., 2020), and lower back pain (Tu et al., 2020). Therefore, brain activity of reading social media posts can be studied by combined fMRI and dFNC analyses.

Several fMRI studies have investigated brain activity while reading social media posts; however, in these studies, the relevant tasks were conducted in the MR scanners, which is not the normal state (Moisala et al., 2016; Sherman et al., 2016; Su et al., 2021). Recent neuroimaging studies show that transient cognitive tasks can result in carryover effects of intrinsic brain activity that can be traced after the task through the functional connectivity (FC) (Gaviria et al., 2021a,b). In brief, the effects of a cognitive task on the cerebral FC may persist for a while, even after the task is terminated. Consequently, we took the advantage of this physiological mechanism to study the brain dynamics of reading social media posts in the natural state.

Although the present study aimed to investigate the influence of social media, we could not preclude the influence of reading activity on the smartphone itself. Thus, we also investigated the brain dynamic activity after reading science fiction on smartphones as the control condition. Science fiction was used as the control because it usually involves a continuous and thought-provoking plot that expands the imagination, thinking, and cognitive abilities, which are assumed to be in contrast from the content consumed through social media (Kidd and Castano, 2013; Scherer, 2009; Hall et al., 2015).

In the current study, we aimed to investigate the brain dynamics of reading social media posts in the natural state. To comprehensively elucidate this issue, we also investigated the brain dynamics of reading science fiction in the natural state as the control. A total of 77 subjects were recruited, and they underwent fMRI scanning at the baseline. Next, the task of reading social media posts or science fiction was performed. Immediately after, the subjects underwent another fMRI scanning procedure. Independent component analysis (ICA), dFNC, graph theory, and FC analyses were combined to investigate the differences between the baseline and after-reading states. The findings may shed light on the brain dynamics of reading social media posts and science fiction and guide the reading habits among the general public.

## Materials and Methods

### Subjects

We recruited a total of 175 subjects from the undergraduate cohort of the Fourth Military Medical University who had responded to a questionnaire with complete demographic information (age, sex, height, weight, family structure, urbanization, ethnic group, smoking habits, drinking habits, color blindness, handedness, and history of traumatic brain injury) and their smartphone-related habits (including time spent on social media, games, chat, movies/TV series, reality shows, novels, and shopping per day). Time spent on social media referred to time spent on the three most common social media apps in China (Microblog, TikTok, and Kwai).

### Overall Study Design

**FIGURE 1:**
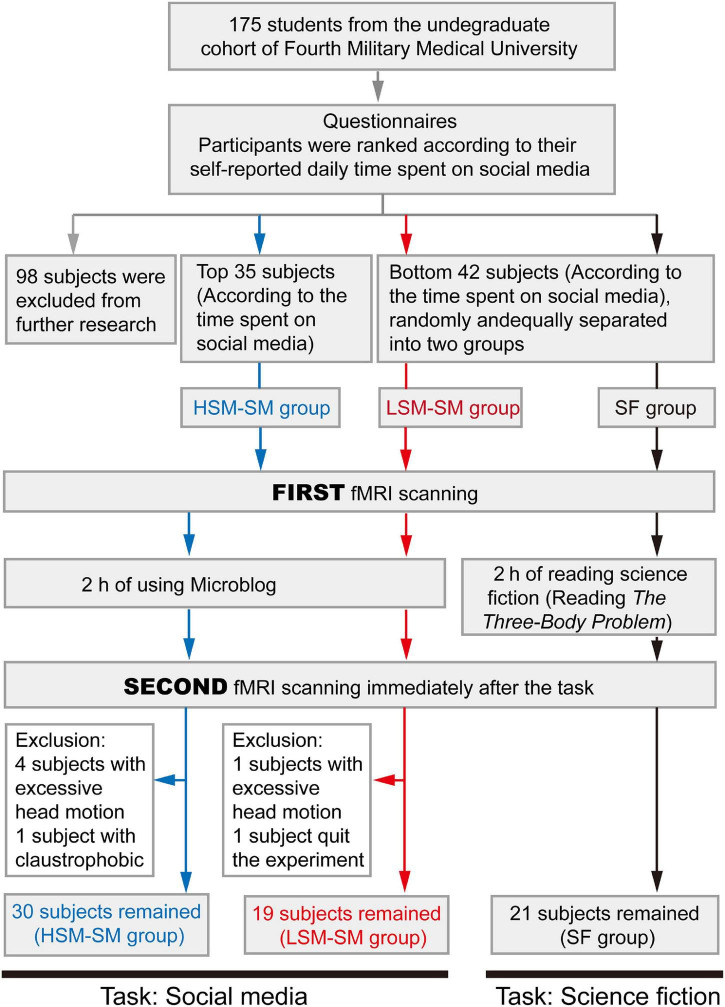
The flow chart for subject recruitment and study design.

Of the 175 subjects who had responded, 77 were recruited based on the amount of time they usually spent on social media per day (Figure 1). Briefly, the top 35 subjects were included in the HSM-SM group (Table 1) to investigate the effects of social media on these subjects with pronounced use of social media. Forty-two subjects with the least use of social media in the cohort were also included (Table 1) and randomly divided into two subgroups (21 subjects each): one to investigate the effect of social media on subjects having less use of social media (defined as the LSM-SM group) and the other to investigate the effect of reading science fiction (defined as the SF group) as the control. Exclusion criteria included a BMI of >30 or <18.5, smoking, alcohol consumption, color blindness, left-handedness, and a history of traumatic brain injury or a family history of mental illness. The experimental design was as follows (Figure 1): (1) subjects underwent a multimodal MRI scan on the first visit; (2) the subjects performed a 2 h reading task (reading social media posts or science fiction); and (3) the subjects underwent a second multimodal MRI scan immediately after the reading task.

**TABLE 1:** Demographic parameters of subjects in the HSM-SM, LSM-SM, and SF groups.

	HSM-SM (*N* = 30)	LSM-SM (*N* = 19)	SF (*N* = 21)	*P*-value
Age (years)	21.13 ± 0.72	20.79 ± 1.01	20.91 ± 1.01	0.495
Height (cm)	175.10 ± 4.25	174.21 ± 6.67	177.41 ± 6.02	0.171
Weight (kg)	67.15 ± 6.31	67.45 ± 7.66	68.93 ± 9.42	0.679
BMI (kg/m^2^)	21.89 ± 1.81	22.18 ± 1.63	21.82 ± 2.08	0.766
**Smartphone use habits**				
Chatting (h)	0.98 ± 0.91	0.87 ± 0.89	1.11 ± 0.85	0.679
Game (h)	0.55 ± 0.52	0.53 ± 0.42	0.68 ± 0.52	0.707
Film/TV series (h)	0.29 ± 0.59	0.21 ± 0.59	0.35 ± 0.57	0.666
Social media (h)	2.05 ± 0.85	0.41 ± 0.36	0.35 ± 0.79	< 0.001*
Novel reading (h)	0.33 ± 1.11	0.39 ± 0.74	0.74 ± 1.07	0.269
Shopping (h)	0.10 ± 0.23	0.06 ± 0.22	0.08 ± 0.23	0.778

*Values are reported as mean ± standard deviation; significant differences are marked with asterisks.*

### Task Design for Reading Social Media Posts

Microblog (Weibo.com) is one of the most widely used social media apps in China with similar functions as Twitter and Facebook. Consequently, surfing Microblog composed the task of reading social media posts; no other apps were allowed to be used during the task to prevent potential biases. The reading tasks were conducted in a quiet room in the laboratory.

### Task Design for Reading Science Fiction

To study the brain dynamics of reading science fiction, 21 subjects in the SF group were included in the science fiction reading task with *The Three-Body Problem* written by *Ci-Xin Liu*, a full-length science fiction with a continuous and thought-provoking plot. To avoid bias due to smartphone use, subjects read an electronic version of the novel on their smartphones. Similar to the social media task, the subjects were not allowed to use other apps during the task.

### Neuroimaging Acquisition and Data Preprocessing

All MRI scans were obtained using the GE Discovery MR750 3.0-T scanner with an eight-channel phased-array head coil. Foam pads were used to limit head movement, and earplugs were used to reduce the scanner’s noise. During the acquisition, participants were told to close their eyes, but not fall asleep. All parameters of neuroimaging data acquisition as listed below were the same as described previously (Hu et al., 2019a,b, 2021).

#### T1-Weighted Imaging Data Acquisition and Processing

We used three-dimensional brain volume (3D-BRAVO) sequences to acquire high-resolution T1-weighted images based on the following parameters: time of echo (TE) = 3.2 ms, time of repetition (TR) = 8.2 ms, flip angle (FA) = 12°, field of view (FOV) = 256 × 256 mm^2^, matrix = 256 × 256, slice thickness = 1.0 mm, and slice number = 188. T1-weighted imaging data were processed using the VBM8 toolkit^1^ with the statistical parameter mapping (SPM12, Wellcome, Imaging Neurology Group, London, United Kingdom^2^) plug-in of MATLAB 2014a. T1 images were spatially normalized to the Montreal Neurological Institute (MNI) space using the diffeomorphic anatomical registration through the exponentiated Lie algebra (DARTEL) algorithm. The Brain Extraction Tool (BET integrated with MRIcro^3^) was used to obtain the brain tissue. The T1 data were used to normalize resting-state blood oxygen level-dependent (BOLD) data to standard space.

#### Resting-State Blood Oxygen Level-Dependent Data Acquisition and Processing

Resting-state BOLD images were acquired using the gradient-recalled EPI (GRE-EPI) sequences with the following parameters: TR = 2,000 ms, TE = 30 ms, FA = 90°, slice number = 36, gap = 3 mm, FOV = 220 × 220 mm^2^, matrix = 64 × 64, slice thickness = 3 mm, in-plane spatial resolution = 3.44 × 3.44 mm^2^, and time points = 185. The BOLD images were preprocessed using the DPABI (Yan et al., 2016) toolkit. The first 10 time points were discarded to ensure magnetization stability and allow for consideration of participant adaptation to the scanning environment. Slice timing and head-motion corrections were performed, and scans with a head motion of translation greater than 2.0 mm or a rotation greater than 2° were excluded. BET was used to extract brain tissue and form BOLD brain images, which were co-registered with the corresponding T1-weighted brain images; the raw BOLD images were also co-registered. We used the transformation field data obtained from T1-weighted image normalization to normalize the co-registered BOLD images to MNI space; subsequently, they were resampled to 3 × 3 × 3 mm^3^ dimensions. The BOLD images were smoothened with an 8-mm full width at half maximum (FWHM) isotropic Gaussian kernel.

#### Independent Component Analysis and Dynamic Function Network Connectivity Analysis

The protocol for ICA and dFNC analysis closely followed that enumerated in previous research on chronic low back pain (Tu et al., 2020). Briefly, the process consisted of the following five steps: (1) group ICA (GICA) was performed to decompose the BOLD data into multiple independent components (ICs); (2) according to a pre-constructed brain network map, each IC was matched to the brain network; (3) the graphic LASSO sliding window method was used to calculate dFNC between networks; (4) the K-means clustering was used to identify dFNC states and their parameters; and (5) the graph-theory characteristics of each dFNC state were analyzed (including global, local, and nodal efficiencies). Other processes included (1) elimination of linear, quadratic, and cubic trends; (2) regression covariates of head movement; (3) de-spiking of outliers; and (4) low-pass filtering with a cutoff frequency of 0.15 Hz.

#### Abnormal Visual Networks in Each Dynamic Function Network Connectivity State

The investigation of the visual networks (VN) in dFNC states included the following four steps. (1) We selected the two ICs of the VN as our regions of interest (ROIs) by plotting spheres around the peak coordinates (radius = 6 mm). (2) We calculated the correlation between the time series of each ROI and all the other voxels in the brain for each sliding window and obtained Fisher’s *r*-to-*z* transformed connectivity map. Given that there were 150 sliding windows in the previous procedure, in this step, we obtained 150 corresponding connectivity maps for each subject and each ROI. (3) The connectivity maps of all sliding windows belonging to the same state were averaged. Herein, we obtained four connectivity maps (corresponding to the four dFNC states) for each subject and each ROI. (4) We averaged the connectivity maps between two ROIs of VN to form the final connectivity map. In this final step, we obtained four connectivity maps for each subject.

### Statistical Analysis

SPSS 20.0 was used for statistical analysis. One-way analysis of variance (ANOVA) was used to compare the demographic information among the three groups. The paired *t*-test was used to compare neuroimaging characteristics that conformed to the Gaussian distribution within the group (baseline and after the reading task), while the nonparametric test was used for non-normal distributions. For the comparison of dFNC parameters and states, a false discovery rate (FDR) correction was used for multiple comparison correction (Tu et al., 2020). In the voxel-wise comparison, the Gaussian random field (GRF) correction was used for multiple comparison correction. The significance levels for the voxel and cluster were set to *P* < 0.005 and *P* < 0.025, respectively, which could effectively control the false-positive rate within 0.05 (Chen et al., 2018).

## Results

### Demographic Information and Smartphone Habits

The demographic information and smartphone habits of the three groups are listed in Table 1. There were no significant differences in demographic parameters and smartphone habits among the three groups, except for the time spent on social media per day (*P* < 0.001).

### Estimation of Whole-Brain Independent Component and Dynamic Function Network Connectivity Analysis

**FIGURE 2:**
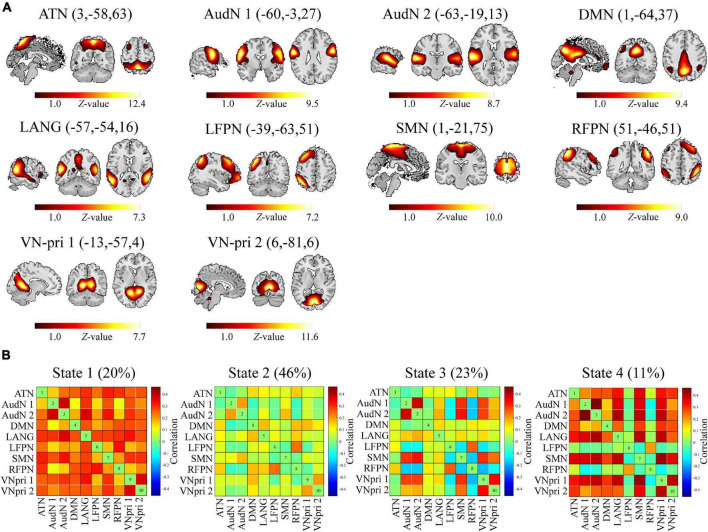
ICs and brain states. **(A)** Ten ICs were derived from group ICA (GICA) and further matched with a previously reported brain network map. **(B)** After K-means clustering for dynamic functional connectivity (FC) between aforementioned ICs, four reoccurring brain states were derived. ATN, Attention network; AudN, Auditory network; DMN, Default mode network; LANG, Language network; LFPN, Left frontal-parietal network; SMN, Somatomotor network; RFPN, Right frontal-parietal network; VN-pri, Primary visual network.

We used the GICA method to decompose the BOLD data into a total of 28 ICs (automatically estimated) and finally identified 10 ICs that matched well with a previously published brain network map (Figure 2A; Iraji et al., 2019). We then calculated the dFNC with a 25 TR window (σ = 3 TRs). K-means clustering was performed with the elbow criterion to generate four reoccurring dFNC patterns (Figure 2B), with the corresponding frequencies of 20, 46, 23, and 11%. All the brain networks were found to be tightly connected in state 1, while in state 2, all brain networks showed loose connections. In state 3, only the auditory network (AudN), somatomotor network (SMN), and VN were tightly connected. State 4 was similar to state 1, except for the left frontoparietal network (LFPN) and the right frontoparietal network (RFPN), which were loosely connected with other networks.

### Impact of Reading Tasks on Dynamic Function Network Connectivity Features

**FIGURE 3:**
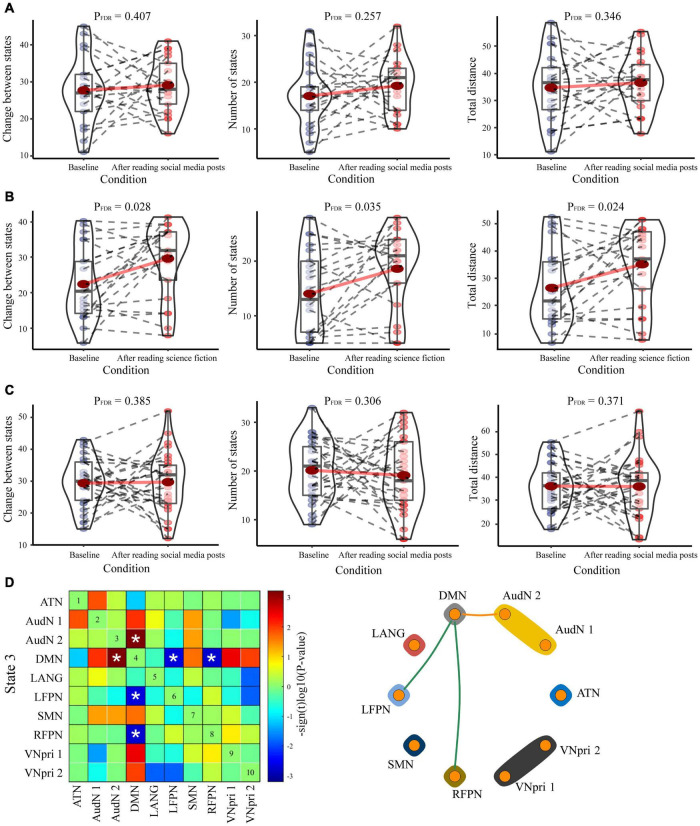
The intragroup comparisons for dFNC parameters. Three dFNC parameters (changes between states, number of states, and total distance) in the baseline and the states after the reading tasks were compared among the **(A)** HSM-SM group, **(B)** LSM-SM group, and **(C)** SF group. Significant differences are labeled with asterisks. **(D)** The comparison of dFNC states within the LSM-SM group at the baseline state and after the reading task. Results have been displayed either as a matrix plot (left, significant results are labeled with asterisks) or as a circle plot (right, only significant results are plotted as edges).

The meta-state dFNC features in LSM-SM (Figure 3A) and HSM-SM groups (Figure 3C) were not significantly altered after the social media post reading task. However, in the SF group, after reading science fiction (Figure 3B), the changes in state (baseline = 21.90 ± 9.66, after reading = 28.81 ± 9.84, *P* = 0.028, FDR corrected), the number of states (baseline = 14.05 ± 7.62, after reading = 18.62 ± 7.02, *P* = 0.035, FDR corrected), and the total distance (baseline = 25.33 ± 12.74, after reading = 33.19 ± 12.61, *P* = 0.024, FDR corrected) showed a significant increase. In state 3, in the LSM-SM group, after the social media post reading task, there was an increase in the FC between the default mode network (DMN) and AudN, along with a decrease in FC between DMN and bilateral FPN (Figure 3D, *P* < 0.05, FDR corrected).

### Impact of Reading Tasks on Dynamic Network Efficiency

**FIGURE 4:**
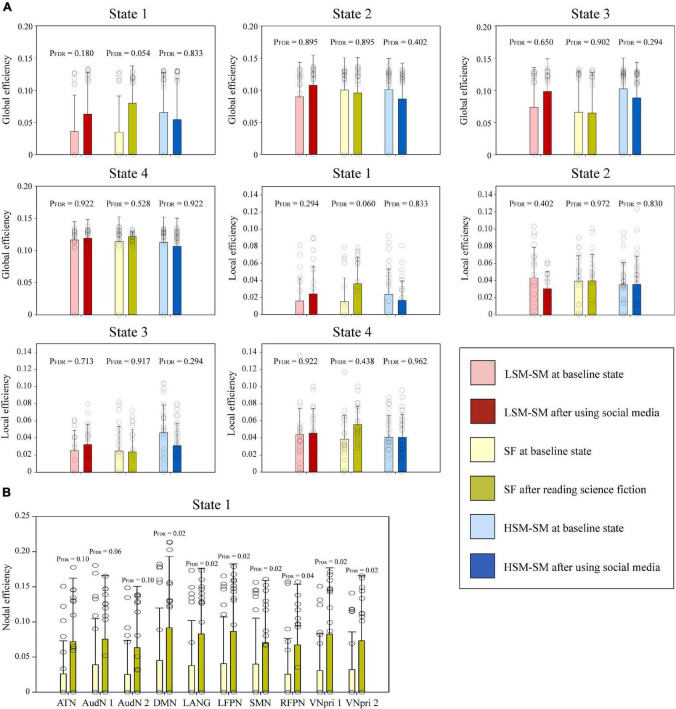
The intragroup comparisons of graph-theory parameters of each dFNC state. **(A)** Two graph-theory parameters (global and local efficiencies) of the baseline and the states after reading tasks were compared among the HSM-SM group, LSM-SM group, and SF group. **(B)** The nodal efficiencies of the 10 networks in the baseline state and after reading science fiction were compared within the SF group. Significant differences are labeled with asterisks.

After the science fiction reading task in the SF group (Figure 4A), the global (baseline = 0.035 ± 0.055, after reading = 0.080 ± 0.057, *P* = 0.054, FDR corrected) and local efficiencies (baseline = 0.015 ± 0.027, after reading = 0.036 ± 0.029, *P* = 0.060, FDR corrected) in state 1 tended to increase. Moreover, the nodal efficiencies of almost all networks of state 1 increased significantly (Figure 4B). However, after the social media post reading task, the changes in network efficiencies were smaller (Figure 4A).

### Abnormalities in the Visual Network

**FIGURE 5:**
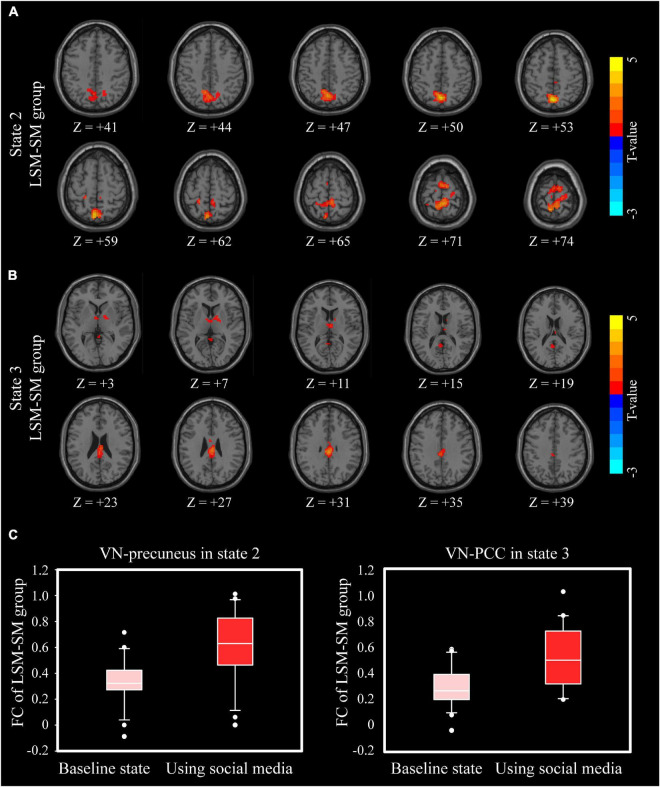
Intragroup differences of VN-whole-brain dFNC. **(A)** In state 2, the differences between baseline and after reading social media posts in the LSM-SM group. **(B)** In state 3, the differences between baseline and after reading social media posts in the LSM-SM group. **(C)** ROI-based FC values in **(A,B)**.

Given that the reading behavior is directly related to visual information processing, we decided to set VN as an ROI to study its dynamic FC with other brain networks. We found that after the social media post reading task, the LSM-SM group showed increased FC between the VN and bilateral precuneus in state 2 (Figure 5A, *P* < 0.05, GRF corrected) as also between the VN and bilateral posterior cingulate cortex (PCC) in state 3 (Figure 5B, *P* < 0.05, GRF corrected). The ROI-based dynamic FC values have been displayed by drawing 6-mm spheres around the peak coordinates in Figure 5C.

### Validation

In our primary experiment, we used the ICA template from Iraji et al. (2019) to assign ICs to the brain networks. We also used two other ICA templates from^4^, the NeuroMark template, and the template from Allen et al. (2011), for network assignment. The results showed that most network assignments were consistent (Supplementary Table 1), especially for those not involved in specific tasks, such as VN, AudN, SMN, and DMN.

We also validated the influence of the Gaussian window alpha value (σ) and window size on the results. In the main experiment, the σ was 3 TRs and the window size was 25 TRs. Consequently, we repeated the dFNC analysis when σ was 1 TR and when the window sizes were 20 TRs and 30 TRs, separately. For dFNC states, all results were consistent with the findings in the main text (Supplementary Figure 1). For dFNC parameters, some differences decreased to the trend levels; however, most results were consistent (Supplementary Table 2).

## Discussion

In this study, we combined the cerebral dFNC analysis with the carryover effect to study the brain dynamics of reading social media posts or science fiction. We found that the changes between dFNC states, the number of dFNC states, and the total distance between all dFNC states increased after reading science fiction. Furthermore, the global, local, and nodal efficiencies of the deep-thinking state tended to increase after reading science fiction. After surfing social media, participants showed decreased FC between DMN and bilateral FPN, while that between DMN and VN increased.

After GICA and cluster analysis, four repeated states were found among all subjects and scans. The first state was a tightly connected one, with a high overall FC value. This state may reflect the process of deep thinking, which requires several or all the brain networks to work together for effective communication. In contrast, state 2 was a loosely connected state with a low overall FC, most likely reflecting the cerebral resting state with no overthinking. State 3 was also loosely connected, but herein, VN, AudN, and SMN were closely connected. This suggested that state 3 may reflect visual and auditory stimuli, as also the finger and eye movements while reading. State 4 was also tightly connected, except for the LFPN and RFPN, which were found to be loosely connected with other networks. The main functions of LFPN are to do with semantic comprehension and working memory, while those of RFPN are in the working memory, reasoning, and abstraction (Smith et al., 2009). Consequently, state 4 may reflect the processes during mind wandering.

After the reading science fiction task, three dFNC parameters were found to increase, including changes between states, the number of states, and the total distance. However, after the reading social media posts task, these parameters were unchanged. The changes between states and the number of states reflect the ability to switch from one state to another. The total distance not only indicates a change between states but also the extent of transition. Previous research suggests that these parameters increase in schizophrenic patients, who are thought to have pathologically increased mental activity (Sanfratello et al., 2019). Besides, studies also report that these parameters decrease in autistic patients, with pathologically decreased mental activity (Fu et al., 2019). These results suggest that reading science fiction makes the brain more active and increases the number of states and transitions, along with the extent of transition.

Network efficiency is the most widely used graph-theory parameter in brain network analysis. It was developed from the transdisciplinary combination between brain network and physics of complex systems (Bullmore and Sporns, 2009). The network efficiency measures the efficiency to transmit information within a specified network. Previous studies report a negative relationship between aging and network efficiency (Madden et al., 2020). Besides, in depression, wherein the main manifestations include apathy and retardation of thinking, the network efficiency also decreases (Li et al., 2017; Yu et al., 2020). We found that after the reading science fiction task, the global, local, and nodal efficiencies of state 1 improved, thereby indicating the enhanced efficiency of thinking. Furthermore, state 1 is regarded as a brain state of deep thinking. Thus, reading science fiction could specifically improve the efficiency of deep thinking.

After the reading social media posts task, there was an increase in the FC between the VN and precuneus in state 2, as also between VN and PCC in state 3 (Figure 5). Precuneus and PCC are key components of DMN, a complex network system with multiple functions (Raichle, 2015). In state 3, in the LSM-SM group, after the social media post reading task, the FC between DMN and bilateral FPN was found to decline (Figure 3D). A major function of the DMN is in mind wandering (Brewer et al., 2011), including thinking about others, thinking about self, remembering the past, and looking forward to the future (Yeshurun et al., 2021). Consequently, the current results suggest that social media could stimulate the VN and induce mind wandering, along with a decrease in the abilities of reasoning and working memory, specifically among those who do not have pronounced social media-related habits.

Social media is known to be related to inattention and mind wandering (Firth et al., 2019). This may be because the content on social media apps is shallow (short, modular, and unrelated to neighboring content) and usually associated with shallow reading behavior, which is negatively related to the ability to focus on a given task (Baron, 2015; Wang et al., 2019). A recent study also shows that shallow reading is a type of inattentive reading, wherein the mind tends to wander (Delgado and Salmerón, 2021). Consequently, our results overlapped with several findings on shallow reading maybe because the reading mode for social media is relatively shallow. Previous neuroimaging research also reports similar findings, including increased brain activation in the DMN and increased coupling between activated DMN and neural pathways underlying auditory and visual processing, as well as the FPN (Su et al., 2021). In contrast, brain activity increases after reading books, and this mainly results from the increased FC between the visual word forming area and the regions related to language, visual processing, and cognitive control (Horowitz-Kraus and Hutton, 2018). In terms of graph theory, there was a positive relationship between the global modularity of an individual’s brain network and reading habit (Bailey et al., 2018). The findings were in line with previous studies which show increased brain activity and topological characteristics after reading science fiction. However, we not only reported changes in imaging characteristics after reading social media posts or science fiction but also confirmed the corresponding brain states using dFNC analysis.

However, our study has some limitations. The sample size was relatively small, especially per group; we plan to recruit more subjects in the future. We used the brain carryover effect to study the brain dynamics after reading social media posts or science fiction, which may not fully reflect the real-time conditions. Therefore, we plan to conduct this experiment along with simultaneous scanning and evaluate the consistency between the two. Due to the severe sex imbalance in the cohort (only 8 females out of 175 subjects and only 3 willing participants in the research), only male subjects were included to prevent any potential bias. This was a tough decision, as females generally tend to use social media more frequently than males (Spiller et al., 2019). Therefore, the inclusion of females in future research will be one of our goals.

## Conclusion

According to the aforementioned evidence, we concluded that science fiction reading was related to increased brain activity and network efficiency, while social media post reading was related to abnormal FCs between DMN, VN, and FPN.

## Data Availability Statement

The raw data supporting the conclusions of this article will be made available by the authors, without undue reservation.

## Ethics Statement

The studies involving human participants were reviewed and approved by The Ethics Committee of Tangdu Hospital of Fourth Military Medical University. The patients/participants provided their written informed consent to participate in this study.

## Author Contributions

WW and G-BC: conceptualization and writing – review and editing. BH and Y-LC: data curation. QS and JZ: formal analysis. Y-LC, BH, and G-BC: funding acquisition. BH: investigation. L-FY and YY: methodology. BH and WW: project administration. J-TS: resources. Y-TL: software. WW and G-BC: supervision. Y-LC: visualization. BH and YY: roles/writing – original draft. All authors contributed to the article and approved the submitted version.

## Conflict of Interest

The authors declare that the research was conducted in the absence of any commercial or financial relationships that could be construed as a potential conflict of interest.

## Publisher’s Note

All claims expressed in this article are solely those of the authors and do not necessarily represent those of their affiliated organizations, or those of the publisher, the editors and the reviewers. Any product that may be evaluated in this article, or claim that may be made by its manufacturer, is not guaranteed or endorsed by the publisher.

## Footnotes

1
http://dbm.neuro.uni-jena.de/vbm/

2
http://www.fil.ion.ucl.ac.uk/spm

3
http://www.mricro.com

4
https://trendscenter.org/data/
