# Supplementary material for: Combining Dynamic Network Analysis and Cerebral Carryover Effect to Evaluate the Impacts of Reading Social Media Posts and Science Fiction in the Natural State on the Human Brain
Source: Front Neurosci. 2022 Feb 21;16:827396. doi: 10.3389/fnins.2022.827396 (PMC8901113; doi:10.3389/fnins.2022.827396)
Supplement: Supplementary file 1 [file Image_1.pdf]

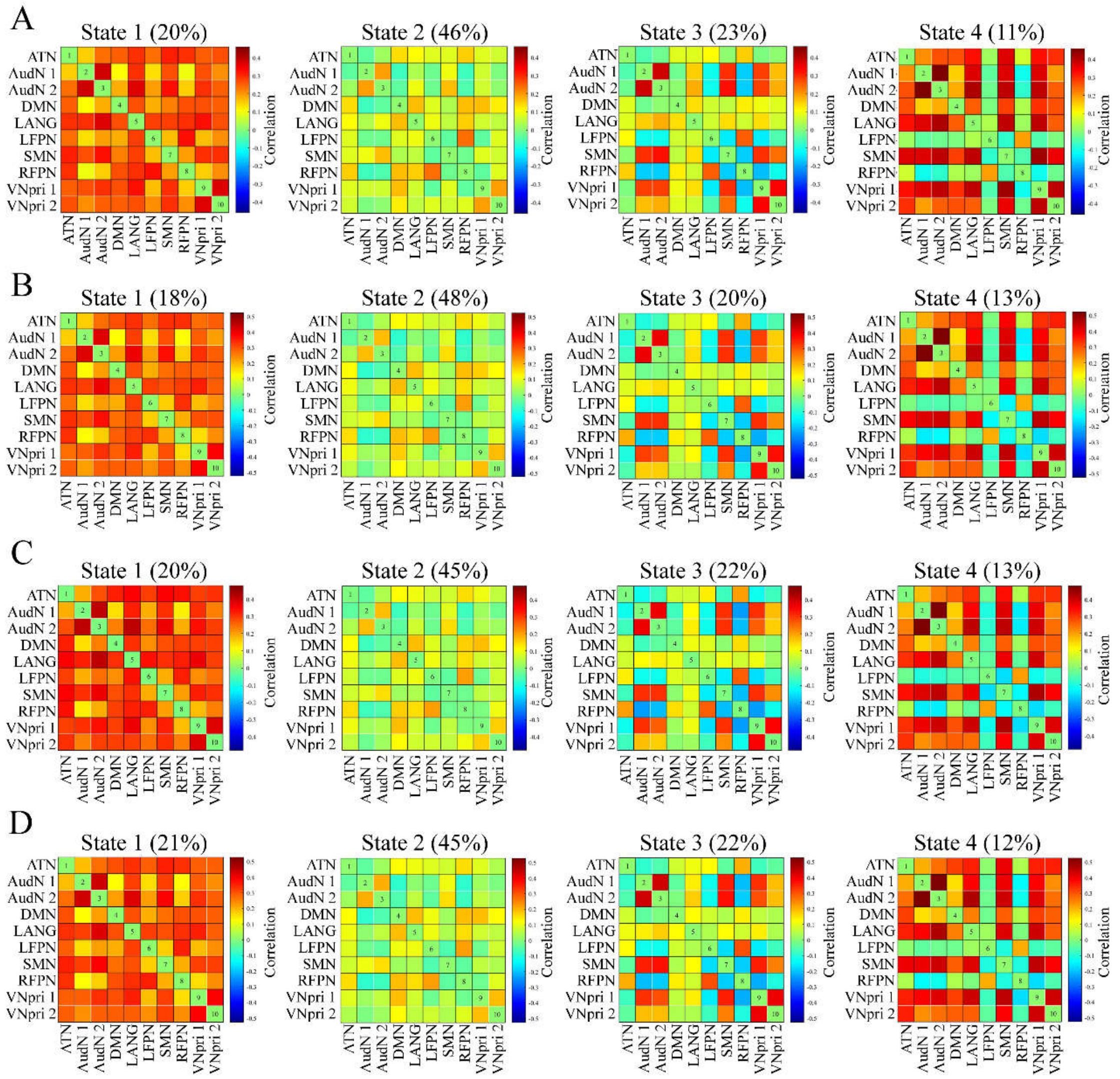

Fig S1. Validation test about the influence of Gaussian window alpha value (σ) on dFNC states. (A) Four dFNC states in the main text, the σ was 3 TRs, and the window size was 25 TRs. (B) Four dFNC states when the σ was 1 TRs, and the window size was 25 TRs. (C) Four dFNC states when the σ was 3 TRs, and the window size was 20 TRs. (D) Four dFNC states when the σ was 3 TRs, and the window size was 30 TRs. We can see that both the states and their frequencies were consistent.
