# Supplementary material for: Combining Dynamic Network Analysis and Cerebral Carryover Effect to Evaluate the Impacts of Reading Social Media Posts and Science Fiction in the Natural State on the Human Brain
Source: Front Neurosci. 2022 Feb 21;16:827396. doi: 10.3389/fnins.2022.827396 (PMC8901113; doi:10.3389/fnins.2022.827396)
Supplement: Supplementary file 2 [file Table_1.docx]

**Table S1**. The validation of independent component analysis and network assignment by using different templates.

| Independent component | PowerLF/PowerHF | Template from Iraji et al. | Neuromark template | Template from E. Allen et al. |
| --- | --- | --- | --- | --- |
| 27 | 5.088 | VN | VN | VN |
| 26 | 2.403 | AudN | AudN | AudN |
| 22 | 6.321 | VN | VN | VN |
| 21 | 7.104 | SMN | SMN | SMN |
| 19 | 5.058 | RPFN | CCN | ATN |
| 14 | 4.460 | LANG | N/A | ATN |
| 08 | 4.305 | DMN | DMN | DMN |
| 06 | 4.187 | AudN | SMN | SMN |
| 04 | 5.304 | LFPN | N/A | ATN |
| 02 | 7.649 | ATN | SMN | ATN |

In the main text, we used the template from Iraji et al. to assign independent components (ICs) into brain networks, here, we used other two templates from <https://trendscenter.org/data/> to validate this assignment, which were the Neuromark template and the template from E. Allen et al. First of all, ICs with PowerLF/PowerHF upper than 2 was regarded as meaningful and selected, and then, templates were used to assign these ICs into brain networks. The results showed that most network assignments were consistent, especially VN, AudN, SMN, and DMN. ATN = Attention network; AudN = Auditory network; DMN = Default mode network; LANG = Language network; LFPN = Left frontal parietal network; SMN = Somatomotor network; RFPN = Right frontal parietal network; VN = Visual network; CCN = Cognitive control network.
