# Supplementary material for: Combining Dynamic Network Analysis and Cerebral Carryover Effect to Evaluate the Impacts of Reading Social Media Posts and Science Fiction in the Natural State on the Human Brain
Source: Front Neurosci. 2022 Feb 21;16:827396. doi: 10.3389/fnins.2022.827396 (PMC8901113; doi:10.3389/fnins.2022.827396)
Supplement: Supplementary file 3 [file Table_2.docx]

**Table S2.** The validation of within-group comparison of independent component analysis parameters by using different Gaussians window alpha value (σ) and window size (WS).

| Statistic values | Paired t-test within HSM group | | | | Paired t-test within LSM group | | | | Paired t-test within SF group | | | | |
| --- | --- | --- | --- | --- | --- | --- | --- | --- | --- | --- | --- | --- | --- |
|  | WS=25TRs  σ = 3 TRs  (main text) | WS=25TRs  σ = 1 TR | WS=20TRs  σ = 3 TRs | WS=30TRs  σ = 3 TRs | WS=25TRs  σ = 3 TRs  (main text) | WS=25TRs  σ = 1 TR | WS=20TRs  σ = 3 TRs | WS=30TRs  σ = 3 TRs | WS=25TRs  σ = 3 TRs  (main text) | WS=25TRs  σ = 1 TR | WS=20TRs  σ = 3 TRs | WS=30TRs  σ = 3 TRs |  |
| Number of states | |  |  |  |  |  |  |  |  |  |  |  |  |
| Baseline | 20.138 | 19.000 | 20.621 | 16.276 | 17.048 | 16.810 | 17.143 | 14.429 | 14.048 | 13.952 | 14.381 | 14.095 |  |
| After task | 19.103 | 19.276 | 20.103 | 16.310 | 19.238 | 18.905 | 19.000 | 16.143 | 18.619 | 17.667 | 18.571 | 16.714 |  |
| *T* value | 0.506 | -0.150 | 0.309 | -0.022 | -1.073 | -1.526 | -1.343 | -1.172 | -2.528 | -1.810 | -2.223 | -1.983 |  |
| *P* value | 0.617 | 0.882 | 0.759 | 0.983 | 0.296 | 0.143 | 0.296 | 0.255 | 0.020 | 0.085 | 0.037 | 0.082 |  |
| Change between states | |  |  |  |  |  |  |  |  |  |  |  |  |
| Baseline | 29.379 | 35.276 | 34.345 | 24.759 | 27.714 | 33.571 | 31.191 | 23.000 | 21.905 | 21.429 | 26.714 | 27.429 |  |
| After task | 29.724 | 35.241 | 34.379 | 25.655 | 29.095 | 33.905 | 31.762 | 24.952 | 28.810 | 27.762 | 30.143 | 32.238 |  |
| *T* value | -0.142 | 0.013 | -0.015 | -0.412 | -0.553 | -0.146 | -0.045 | -0.921 | -2.942 | -2.671 | -1.423 | -1.886 |  |
| *P* value | 0.888 | 0.990 | 0.988 | 0.680 | 0.687 | 0.885 | 0.836 | 0.358 | 0.008 | 0.015 | 0.170 | 0.074 |  |
| Total distance | |  |  |  |  |  |  |  |  |  |  |  |  |
| Baseline | 34.828 | 42.621 | 40.310 | 28.000 | 32.333 | 40.810 | 36.143 | 26.000 | 25.333 | 24.619 | 30.905 | 31.381 |  |
| After task | 34.690 | 42.793 | 40.276 | 29.345 | 33.952 | 41.667 | 37.105 | 28.000 | 33.191 | 32.143 | 35.524 | 38.476 |  |
| *T* value | 0.047 | -0.050 | 0.011 | -0.520 | -0.535 | -0.278 | -0.322 | -1.687 | -2.512 | -2.499 | -1.506 | -2.123 |  |
| *P* value | 0.963 | 0.961 | 0.991 | 0.607 | 0.599 | 0.784 | 0.609 | 0.107 | 0.021 | 0.023 | 0.148 | 0.046 |  |
